# Adequate Management of Phosphorus in Patients Undergoing Hemodialysis Using a Dietary Smartphone App: Prospective Pilot Study

**DOI:** 10.2196/17858

**Published:** 2021-06-01

**Authors:** Cosette Fakih El Khoury, Rik Crutzen, Jos MGA Schols, Ruud JG Halfens, Mirey Karavetian

**Affiliations:** 1 Department of Health Services Research Care and Public Health Research Institute, Faculty of Health, Medicine and Life Sciences Maastricht University Maastricht Netherlands; 2 Department of Health Promotion Care and Public Health Research Institute, Faculty of Health, Medicine and Life Sciences Maastricht University Maastricht Netherlands; 3 Care and Public Health Research Institute Department of Health Services Research Maastricht University Maastricht Netherlands; 4 Department of Health Sciences Zayed University Dubai United Arab Emirates

**Keywords:** renal diet, mhealth, dietary app

## Abstract

**Background:**

The renal diet is complex and requires alterations of the diet and careful monitoring of various nutrients. Elevated serum phosphorus is common among patients undergoing hemodialysis, and it is associated with many complications. Smartphone technology could be used to support both dietitians and patients by providing a source of accessible and reliable information.

**Objective:**

The aim of this pilot is to assess the potential efficacy of an intervention using the educational and self-monitoring mobile app KELA.AE on the phosphorous management in hemodialysis patients. Results will be used to improve both the app and a planned, rigorous large-scale trial intended to assess app efficacy.

**Methods:**

This is a prospective pilot study performed at the hemodialysis unit of Al Qassimi Hospital (Emirate of Sharjah, United Arab Emirates). All patients were assessed for eligibility and, based on inclusion criteria, considered for enrollment. Participants met with a dietitian once a week and used the mobile app regularly for 2 weeks. Outcomes (knowledge, self-reported nonadherence, dietary intake, anthropometry, and biochemical data) were measured. This pilot is reported as per guidelines for nonrandomized pilot and feasibility studies and in line with the CONSORT (Consolidated Standards of Reporting Trials) 2010 checklist for reporting pilot or feasibility trials.

**Results:**

Of 26 subjects, 23 successfully completed the pilot. Patient dietary knowledge about phosphorous management improved from 51.4% (SD 13.9) to 68.1% (SD 13.3) after intervention with a large effect size (*d*=1.22, 95% CI 0.59 to 1.85). Dietary protein intake increased from a mean of 0.9 g/kg (SD 0.3) per day to a mean of 1.3 g/kg (SD 0.5) per day with a large effect size (*d*=1.07, 95% CI 0.45 to 1.69). Phosphorus to protein ratio dropped from a mean of 18.4 mg/g protein to 13.5 mg/g protein with a large effect size (*d*=0.83, 95% CI 0.22 to 1.43). There was no evidence of change in phosphorous intake, self-reported nonadherence, and serum phosphorus.

**Conclusions:**

The findings of this prospective pilot reveal the potential efficacy of a smartphone app as a supportive nutrition education tool for phosphorus management in patients undergoing hemodialysis. This pilot study showed that the KELA.AE app has the potential to improve knowledge and dietary choices. A rigorous randomized controlled trial should be performed to evaluate the efficacy, assessing app use of a long-term intervention.

## Introduction

Dietary management plays an essential role in slowing down disease progression and improving the quality of life of people with chronic kidney disease (CKD) [1,2]. However, the renal diet is complex and requires targeted attention of the intake of various nutrients [3], and patients often express frustration and confusion about this [4]. Dietary adjustments are made continuously in response to alterations in blood parameters, and patients must be constantly monitored and followed up accordingly [5].

Elevation in blood phosphorus is specifically common among this patient group, and it is associated with many comorbidities such as cardiovascular disease, metabolic bone mineral disease, and mortality [5]. Even a mild hyperphosphatemia (greater than 5.0 mg/dL) is independently associated with an increase in mortality among hemodialysis patients [6]. Therefore, management of hyperphosphatemia is essential during hemodialysis; however, this seems to be particularly challenging. Patients are recommended to consume 1.2 g/kg body weight of protein to achieve protein needs during hemodialysis [3]. Moreover, phosphorus is typically found in protein-rich foods; thus, restricting dietary phosphorus to the recommended 800 to 1000 mg/d while maintaining adequate protein intake is the cornerstone of this diet [3]. Such recommendations are not easily compatible [7] and lead to conflicting and ambiguous information that requires simplification and clarification from the dietitian’s side [8].

Adherence to the renal diet is, therefore, an essential component for the management of hemodialysis patients [9]. Patient knowledge can play a role in compliance with the diet [10]. Therefore, nutrition education and counseling might have a positive effect on blood phosphorous [11]. However, eating habits are complicated and not merely influenced by knowledge, but also by a patient’s readiness to change and their health beliefs [12]. The association between knowledge and adherence is not always clear, and an increase in knowledge does not necessarily lead to improved adherence [13]. Nutrition education can, nevertheless, improve nutrition knowledge, which, in turn, can support increased dietary adherence [14,15]. This knowledge is especially valuable when patients are willing to change their dietary behaviors [13].

From the patient’s perspective, receiving easy to understand individualized nutrition education at the early stages is desired [4]. Educational materials should be theory-based and adapted to the patient [2] to be effective. A thorough education has been listed among the facilitators for improvements of serum phosphorus. Such interventions consist of multiple long encounters with patients and thus may not always be feasible [16]. Time limitation during encounters with dietitians and physicians often act as a barrier to effective nutrition education [17].

A recent study qualitatively explored the experience of renal dietitians. Dietitians expressed frustration, limited resources, and emotional and professional challenges in providing dietary education to CKD patients [8]. Hemodialysis patients show similar frustration and a need for continuous access to reliable nutrition information. Therefore, alternative approaches should be explored that may support both dietitians and patients in overcoming these difficulties. Smartphone technology can provide persons with chronic diseases with accessible and reliable information [18]. Additionally, in-app educational materials allow patients access to nutrition education in different modalities [19]. Accordingly, dietary apps may be effective at improving nutritional outcomes in chronic diseases [20].

Freely accessed educational websites are available to CKD patients, but they target persons with high computer literacy [21]. Commercially, some renal nutrition apps are also available, but they are mostly available in the English language only and may include a subscription fee [22,23]. Research in this area is still scarce as only a few publications have addressed the effectiveness of mHealth in the context of CKD [23]. Additionally, only a few registered clinical trials are active in the area of mHealth in CKD [24].

The IDEAS (integrate, design, assess, and share) framework for the development of effective digital interventions defines the process of assessing the efficacy of a developed product as starting with a pilot study aiming at estimating potential efficacy. Information gathered from the pilot study would then be used to improve the product itself and the study design of a rigorous randomized controlled trial (RCT) [25].

This is a pilot study that aims to explore the potential efficacy of an intervention using a smartphone app in the phosphorus management of patients undergoing hemodialysis. The results of this pilot study will be used to improve the app itself along with the study design of a rigorous RCT. The app used is research-based and includes self-monitoring features, educational features, and CKD-friendly recipes in English and Arabic. The description of the person-centered and theory-based app development is detailed in a separate publication [19].

## Methods

### Study Design

This is a prospective pilot study conducted using a theory-based educational dietary app Kidney Education Lifestyle Application (KELA.AE). This pilot followed the guidelines for reporting nonrandomized pilot and feasibility studies [26]. This study is also reported in line with the CONSORT (Consolidated Standards of Reporting Trials) 2010 checklist of information to include when reporting a pilot or feasibility trial excluding items pertinent to randomization (Multimedia Appendix 3) [26,27]. Outcome measures include knowledge, self-reported nonadherence, dietary intake, anthropometry, and biochemical data.

### Participants, Eligibility, and Recruitment

A list of all patients undergoing hemodialysis was obtained from the hemodialysis unit of Al Qassimi Hospital (Emirate of Sharjah, United Arab Emirates), and all subjects were approached during their scheduled dialysis session to identify those who met the inclusion criteria. The study was explained to eligible participants, and signed consent forms were collected from those who agreed to participate. A total of 26 participants were recruited. Patients undergoing hemodialysis for at least 3 months; free of life-threatening conditions; able to read, write, listen, and communicate in Arabic; owning an Android smartphone; and not having been hospitalized in the past 6 months were eligible to participate in the study. Recruitment and data collection began in February 2019 and ended in April 2019. Post hoc calculations of sample size in pilot studies, assuming detection of unanticipated problems with a probability of at least 15% (π=0.15) and a 95% confidence level, resulted in a required sample of 19 subjects [28].

### Ethical Approvals

The study received Institutional Review Board approval (ZU17_066_F) from Zayed University, Dubai.

### Procedure

Participants were provided with a username to initiate the sign-in procedure. Upon registration, participants were provided with a brief orientation to the app features, and free access to KELA.AE was provided for 2 weeks. During this period, participants met face-to-face with a research dietitian once a week. The dietitian provided participants with reinforcements of the critical messages relayed by the educational materials and answered questions about the app use and content. The dietitians also collected data from patients before and after app use. Baseline and postintervention outcomes were assessed before app registration (T0) and after the completion of 2 weeks of app use (T1). The trial was stopped when all participants completed 2 weeks of app use. Participants could keep using the app if they wished; however, all data collection was completed 2 weeks after app use.

### KELA.AE App

The app consists of an Arabic, theory-based, and culture-specific Android app (KELA.AE). A formative study of the app has been published elsewhere [19]. The app was designed to provide dietary education and traditional renal diet–friendly recipes to hemodialysis patients. The transtheoretical model [29] and constructs from the reasoned action approach [30] were incorporated in the development of the educational materials. Three different stages of change have been included along with the concepts of self-efficacy, norms, and attitudes. Behavior change techniques used included self-monitoring of behavior, problem solving and coping planning, goal setting, social comparison of behavior, and verbal persuasion to boost self-efficacy. The stages and constructs were incorporated based on previously published, validated stage-based materials [31], qualitative data, and brainstorming sessions performed during app development by the research team [19]. Different educational modalities were used to deliver the behavior change techniques: notifications, podcasts, and videos. Each modality is matched to a behavior stage and a construct from the reasoned action approach; an example would be a notification that is delivered to the user stating “Many dialysis patients have benefited from the advantages of exercising and were able to improve their quality of life. You can do it too!” (this is provided to a patient categorized in the action stage and linked to capacity/self-efficacy). Additional examples of educational materials are reported in a separate publication [19]. Self-monitoring features to track food intake and blood tests are also available.

The app was developed as a collaboration between the research team and the design team of an app development company. The technical development of the app software was outsourced by the research team to the development company. Educational materials were prepared by the research team in collaboration with the departments of Arabic and communications of Zayed University. The app is hosted on the servers of the development company.

### Self-Reported Nonadherence

The Dialysis Diet and Fluid nonadherence Questionnaire was used to assess self-reported dietary nonadherence [32] (Multimedia Appendix 1). The questionnaire includes 2 simple questions on the frequency and degree of nonadherence. The same questions are asked for overall dietary nonadherence and again for dietary phosphorus nonadherence (total of 4 questions). It requires the patient to report nonadherence as the number of nonadherent days in the last 14 days. The degree of nonadherence is reported on a Likert scale (0 to 4, where 0 means compliant and 4 means severe nonadherence). The questions were translated to Arabic but given their straightforward simplicity further validation was deemed unnecessary. Construct validity of the original tool was reported using a Kendall tau correlation for frequency and degrees of nonadherence (τ=0.495; *P*<.001) [32]. Adherence via the app was not collected due to the lack of in-app analytics in the current version of the app.

### Knowledge

The Knowledge Questionnaire consists of 18 questions assessing knowledge about the renal diet (Multimedia Appendix 2). A total of 18 points can be achieved if all answers are correct; scores were then converted into a percentage. This tool was used in Arabic before [33] based on an adaptation of the original questionnaire [34] and now includes Arabic foods. A score of 60% in overall knowledge (all 18 questions) was considered as sufficient knowledge as recommended by the questionnaire [33]. Questions were clustered by topic, and a subanalysis was performed to understand knowledge in specific areas pertinent to phosphorous management (phosphorus content in food, consequences of hyperphosphatemia, and use of phosphate binders).

### Dietary Intake

Dietary intake was assessed by 2 trained research dietitians using face-to-face 24-hour recalls [35]. Participants were asked if the day before was deemed representative of the previous week, and if not, they were asked to report a typical day for better representation of the past week’s intake. The 24-hour recalls were analyzed using the FoodData Central of US Department of Agriculture databases [36]. Phosphorus needs were considered as 1000 mg/d for participants with serum phosphorus below 5.5 mg/dL and 12 mg/g of protein intake for participants with serum phosphorus below 5.5 mg/dL [37].

### Biochemical Parameters

Blood parameters were retrieved from patient medical records as part of the routine protocols of the hemodialysis unit (measurements are taken post hemodialysis session). Target values for serum phosphorus in hemodialysis were considered between 3.5 and 5.5 mg/dL based on the National Kidney Foundation Kidney Disease Outcomes Quality Initiative (NKF KDOQI) recommendations [3].

### Anthropometric Measurements

Body weight and height were retrieved from patient medical records, as measured postdialysis routinely. BMI was calculated accordingly using measured body weight and height. Comparative standards for body weight were used as suggested by the Nutrition Care Manual [37] based on the NKF KDOQI guidelines [3]. Accordingly, standard body weight from the National Health and Nutrition Examination Study was used for the calculation of nutrient needs. Adjusted edema-free body weight was used for the calculation of nutrient needs for participants with <95% or >115% of standard body weight [37] as recommended by the guidelines.

### Statistical Analysis

SPPS (version 21, IBM Corp) was used to perform all statistical analyses. Categorical variables were described using frequencies and percentages, while means and standard deviations were used to represent continuous variables. A Shapiro-Wilk normality test was performed to ensure that data are normally distributed. Paired *t* tests were used to compare the mean scores before and after the intervention. Two-tailed *P* values are reported. Effect sizes were calculated as Cohen *d* (with 95% CI) using mean difference and pooled standard deviations. The effect size was considered small at 0.2, medium at 0.5, and large at 0.8 and above [38].

## Results

**Figure 1:**
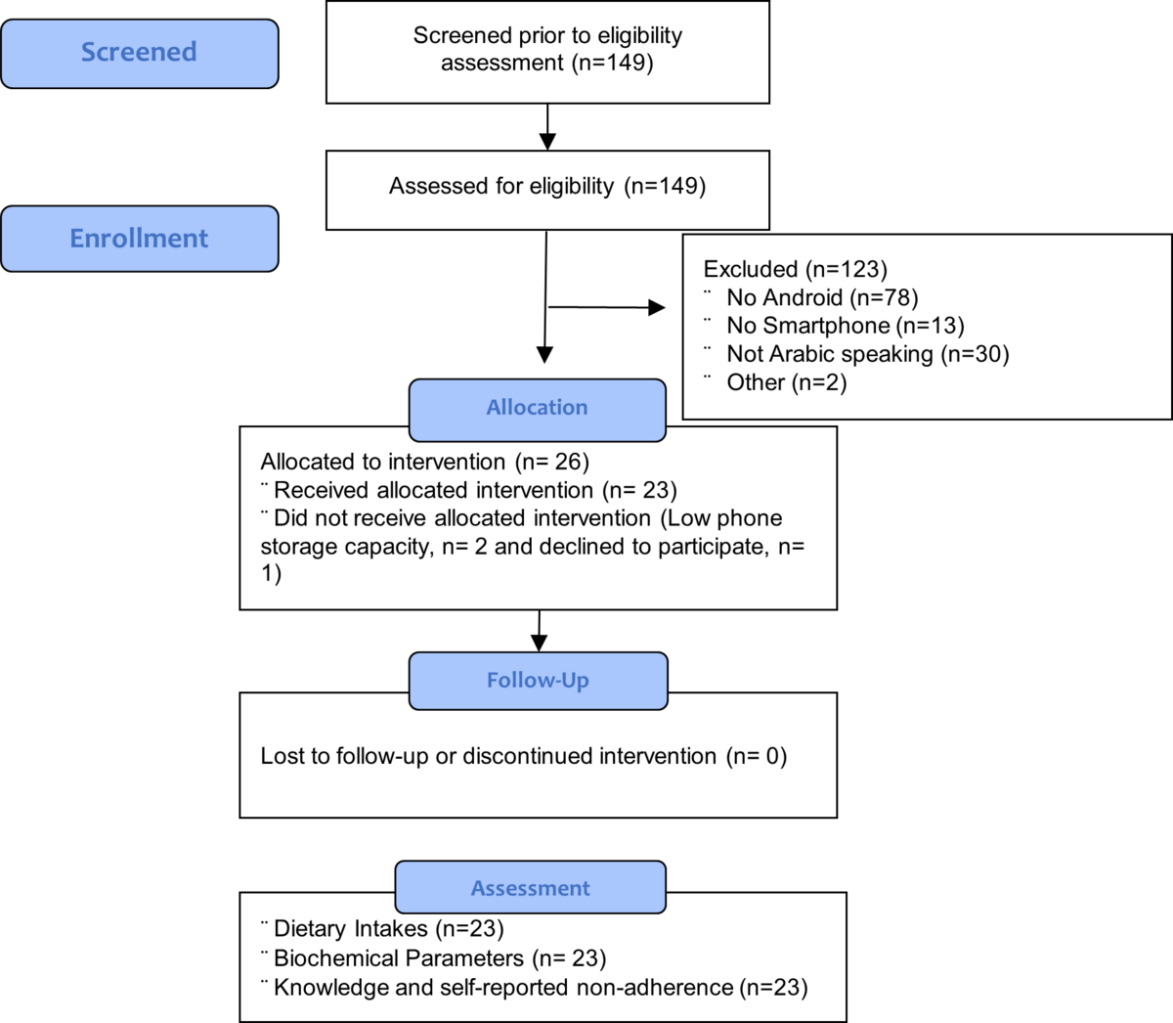
CONSORT study flow diagram.

A total of 23 participants completed the app pilot testing. Two subjects were excluded due to issues related to low smartphone storage capacity, and one subject was not interested in downloading the app. The mean age of the participants was 48.5 (SD 13.7) years, mean BMI was 31.9 (SD 7.9) kg/m^2^, and mean time on dialysis was over 1 year, with 29.7 (SD 37.3) months of dialysis. More men were enrolled in the study (14/23, 61%), and most participants suffered from hypertension (16/23, 70%) or diabetes (11/23, 48%). Demographics measured at baseline are shown in Table 1. Figure 1 depicts the CONSORT flow diagram.

**Table 1:** Demographics and baseline characteristics of the study population (n=23).

Characteristic			Value
Age (years), mean (SD)			48.5 (13.7)
BMI (kg/m^2^), mean (SD)			31.9 (7.9)
Months on dialysis, mean (SD)			29.7 (37.3)
**Gender, n (%)**			
	Male	14 (61)	
	Female	9 (39)	
Smoker, n (%)			6 (26)
**Comorbidities, n (%)**			
	Hypertension	16 (70)	
	Diabetes	11 (48)	
	Dyslipidemia	2 (9)	
	Cancer	1 (4)	
	Liver disease	1 (4)	

### Knowledge and Nonadherence

Overall, the mean self-reported nonadherence days dropped from 3.2 (SD 4.5) over the past 14 days to 2.0 (SD 3.0) days after exposure to in-app education with a small effect size (*d*=0.33, 95% CI –0.25 to 0.91). The severity of nonadherence was mostly mild and moderate preintervention and postintervention.

The mean perceived days of nonadherence to the phosphorus content of the diet increased from 1.1 (SD 3.2) days to 1.9 (SD 3.0) days with a small effect size (*d*=0.25, 95% CI 0.33 to 0.83). The severity of nonadherence changed from 19 participants reporting no deviation from the diet to only 10 reporting no deviation.

Knowledge was below the 60% cutoff point of adequate knowledge at baseline and improved to reach a mean of 68.1% (13.3) after intervention, with a large effect size (*d*=1.22, 95% CI 0.59 to 1.85). Specific knowledge pertinent to consequences of hyperphosphatemia and phosphate binders also improved (large effect sizes *d*=1.15, 95% CI 0.53 to 1.77, and *d*=1.00, 95% CI 0.39 to 1.61, respectively). Knowledge about the phosphorus content of food improved, with a medium effect size (*d*=0.54, 95% CI –0.05 to 1.12). Results pertinent to adherence and knowledge are detailed in Table 2.

**Table 2:** Baseline and postintervention self-reported dietary nonadherence and dietary knowledge (n=23).

Questionnaire			Baseline	Postintervention	Cohen *d* (95% CI)	*P* value
**DDFQ^a^, overall**						
	Nonadherence (days), mean (SD)		3.2 (4.5)	2.0 (3.0)	0.33 (–0.25 to 0.91)	.32
	**Nonadherence (degree), n (%)**					
		No deviation (0)	11 (48)	11 (11)	—^b^	—
		Mild (1)	5 (22)	4 (4)	—	—
		Moderate (2)	4 (17)	7 (7)	—	—
		Severe (3)	2 (9)	1 (1)	—	—
		Very severe (4)	1 (4)	0 (0)	—	—
**DDFQ, phosphorus**						
	Nonadherence (days), mean (SD)		1.1 (3.2)	1.9 (3.0)	0.25 (0.33 to 0.83)	.45
	**Nonadherence (degree), n (%)**					
		No deviation (0)	19 (83)	10 (44)	—	—
		Mild (1)	1 (4)	5 (22)	—	—
		Moderate (2)	2 (9)	7 (30)	—	—
		Severe (3)	0 (0)	1 (4)	—	—
		Very severe (4)	1 (4)	0 (0)	—	—
**KnQ^c^, mean (SD)**						
	% Overall knowledge (>18 questions)		51.4 (13.9)	68.1 (13.3)	1.22 (0.59 to 1.85)	<.001
	Knowledge of phosphorus content of food (>7 questions)		47.8 (21.4)	57.1 (12.2)	0.54 (–0.05 to 1.12)	.06
	Knowledge of consequences of high levels of phosphorus (>4 questionnaire)		43.5 (18.8)	66.3 (20.8)	1.15 (0.53 to 1.77)	<.001
	Knowledge of phosphate binders (>4 questions)		48.9 (29.6)	76.1 (24.4)	1.00 (0.39 to 1.61)	<.001

^a^DDFQ: Dialysis Diet and Fluid Questionnaire.

^b^Not applicable.

^c^KnQ: Knowledge Questionnaire.

### Serum Phosphorus and Dietary Intake

Dietary protein intake increased from a mean intake of 0.9 (SD 0.3) g/kg per day to a mean intake of 1.3 (SD 0.5) g/kg per day with a large effect size (*d*=1.07, 95% CI 0.45 to 1.69). Phosphorus intake as compared to phosphorus needs did not change. However, the phosphorus to protein ratio dropped from a mean of 18.4 mg/g of protein to 13.5 mg/g of protein with a large effect size (*d*=0.83, 95% CI 0.22 to 1.43). This result is desirable given that it is closer to the recommended 12 mg/g of protein.

No changes were identified in serum phosphorus; however, the number of participants with serum phosphorus above 6 mg/dL increased to become 10 subjects as compared to 6 at baseline. This may be in line with the increase in protein intake and a slight increase in total phosphorus intake. Table 3 illustrates data on serum phosphorus and dietary intake.

**Table 3:** Baseline and postintervention dietary intake and serum phosphorus level (n=23).

Parameter			Baseline		Postintervention		Cohen *d* (95% CI)		*P* value
**Phosphorus intake, mean (SD)**									
	Dietary phosphorus (mg/d)	1152.5 (489.8)		1343.1 (83.0)		0.42 (–0.15 to 1.01)		.88	
	% compliance to phosphorus needs	108.3 (44.5)		109.4 (45.5)		0.02 (–0.55 to 0.60)		.72	
	Dietary protein (g/d)	71.1 (26.4)		103.8 (37.8)		1.00 (0.38 to 1.61)		<.001	
	Dietary protein (g/kg/d)	0.9 (0.3)		1.3 (0.5)		1.07 (0.45 to 1.69)		<.001	
	Phosphorus to protein ratio (mg/g)	18.4 (7.9)		13.5 (2.9)		0.83 (0.22 to 1.43)		.01	
Serum phosphorus (mg/dL), mean (SD)			5.3 (1.5)		5.5 (2.0)		0.15 (–0.43 to 0.73)		.60
**Nonadherence to serum phosphorus, n (%)**									
	< 5 mg/dL	11 (48)		11 (48)		—^a^		—	
	5-6 mg/dL	6 (26)		2 (9)		—		—	
	> 6 mg/dL	6 (26)		10 (43)		—		—	

^a^Not applicable.

## Discussion

### Principal Findings

The main findings of this prospective pilot study show that in-app nutrition education, as a supportive tool to dietitians, can improve knowledge of the renal diet among patients undergoing hemodialysis. Thus, the in-app educational features (notifications, podcasts, videos, and recipes) of the KELA.AE app might have potential as a useful source of nutrition education for patients undergoing hemodialysis. The use of smartphones as supportive tools to deliver education may help overcome the time limitation barrier that is reported during face-to-face encounters with dietitians and physicians [17]. Accordingly, app availability may be beneficial to both patients and health care practitioners as supportive tools for regular care.

However, self-reported adherence to the renal diet was not changed after the use of the KELA.AE smartphone app. Self-reported dietary questionnaires may be a source of bias related to social desirability. Nevertheless, adherence to phosphorus intake (24-hour recalls) and serum phosphorus did not improve either. Knowledge scores were not satisfactory at baseline (below the 60% cutoff used) and increased significantly to reach a mean score of 68.1%. It seems that the association between knowledge and dietary nonadherence in dialysis patients is not always clear [39]. In the results of this prospective pilot, nonadherence to the phosphorus content of the diet increased after the intervention. The increase in knowledge may explain this. Patients might have become more aware of the phosphorus content of food and therefore, their self-perception of nonadherence changed accordingly. A study performed in patients with heart failure reported that despite knowledge on the sodium restriction diet being high, only 40% of participants were adherent to the restrictions based on urine sodium excretion. However, perception of the benefits of the diet was correlated with dietary adherence [40]. Nevertheless, other studies have found that intense nutrition education [41,42] and dietary knowledge [15] improve adherence. Accordingly, longer exposure to continuous education both by dietitians and in-app education material may be needed to understand if increased knowledge may improve adherence and possibly serum phosphorus.

An increase in dietary protein was nevertheless observed as an outcome of the intervention. Protein intake in hemodialysis is essential for the prevention of malnutrition. There is also an association between reduced protein intake and increased mortality [43]. The participants in this pilot study started with low protein intake as compared to the needs of ≥1.2 g/kg/d recommended by NKF KDOQI guidelines [3] and achieved an average intake in line with recommendations postintervention. The contradiction between the protein and phosphorous recommendations of the dialysis diet may lead to protein restriction as a result of decreased phosphorous intake. Baseline and postintervention phosphorus intake were within phosphorus recommendations. Therefore, the increase in protein intake did not negatively impact phosphorus intake. Additionally, the phosphorus to protein ratio postintervention was closer to the recommended ratio of 10 to 12 mg/g of protein [3], meaning that patients increased their protein intake while choosing foods that were lower in phosphorus. These dietary changes may be a result of increased patient knowledge. However, the duration of the intervention may have been too short to detect changes in serum phosphorus, which is a limitation of the pilot. This will be better explored in the future trial.

Adherence to phosphate binders is also an essential component in the phosphorous management of dialysis patients. In this pilot, knowledge pertinent to phosphate binders improved postintervention; however, adherence to binders was not measured directly. Additionally, types of binders and their prescriptions were not investigated and benchmarked with recommendations.

### Limitations

Another limitation of the pilot study is the lack of app use data, which was not retrievable due to the lack of in-app analytics. The first and last access for each user are the only data available, and all users had accessed the app at least once during each week. The next version of the app will include in-app analytics to allow the future trial to track if app use influences improvement in outcomes.

Additionally, user acceptability should also be further assessed. A questionnaire was used during this pilot study to explore acceptability; however, all participants provided answers indicating that they strongly agree with all the questions. The team considered the data collected unsuitable for the assessment of acceptability. Acceptability will be assessed in the future trial using the validated Arabic version of the Mobile Application Rating Scale [44]. Qualitative data will also be added to the assessment during the future trial.

Valid interpretation, translation, and generalizability of mHealth interventions also depend on the assessment of treatment fidelity [45]. The future trial will also assess treatment fidelity in-depth to ensure integrity, reliability, and validity of this mHealth intervention before the interpretation and generalizability of the results. During this pilot, we were unable to ensure compliance with all the goals of treatment fidelity proposed by the Treatment Fidelity Workgroup of the National Institutes of Health Behavior Change Consortium [46]. Among the goals that we were able to meet during the pilot are the standardization of treatment and prevention of contamination, participants’ ability to use the app, and provider training. The steps included automated notifications delivered equally to all participants, an orientation session for participants, pilot testing of educational materials, and the training provided to the dietitians meeting patients weekly. However, due to the lack of in-app analytics, we were unable to track the enactment of the self-monitoring tools adequately.

The intervention included encounters with dietitians that may have influenced the outcomes. This methodology was opted as this is how the app use is envisioned in the practice setting. The app would be a tool that dietitians use to support patient education and self-monitoring. The reinforcement of the dietitians, however, may have influenced the results. Accordingly, the future trial will include a control group whereby dietitians see patients with the same frequency but without app use.

This pilot aimed to assess and refine the methodology and procedures of a future trial aiming to detect the efficacy of app use in the adequate management of phosphorous in patients undergoing hemodialysis. Similar to the few available studies evaluating dietary apps for CKD on a smartphone [23], our results show potential benefits on dietary intake. However, this study is unique for evaluating the potential of apps as educational tools to support dietitians. Additionally, it is the first intervention reporting the role of dietary apps for CKD in Arabic. Based on the findings, the planned trial should be designed to compare regular dietary interventions with dietary interventions supported by the app to avoid the possible confounding effects of the dietitian. During this study, participants often consulted with the research team throughout the regular weekly visits; thus, it would be important to explore further if the app enhances the phosphorous management or if it is instead the frequent dietary follow-up that leads to improvements. In conclusion, the future trial should also evaluate the use of phosphate binders, app use data, acceptability, and fidelity, and it should be designed in a way to detect if the app provides any advantages in the clinical outcomes of phosphorous management as compared to intensive dietary follow up by a dietitian.

### Conclusions

The findings of this pilot study indicate that there is potential in the use of a smartphone app as a supportive nutrition education tool. A rigorous RCT should be performed to evaluate the efficacy, in which app use and long-term impact should be assessed.

## Appendix

Multimedia Appendix 1 Dialysis Diet and Fluid non-adherence Questionnaire.

Multimedia Appendix 2 Knowledge Questionnaire.

Multimedia Appendix 3 CONSORT 2010 checklist of information to include when reporting a pilot or feasibility trial.

## Abbreviations

CKD: chronic kidney disease
CONSORT: Consolidated Standards of Reporting Trials
IDEAS: integrate, design, assess, and share framework
KELA.AE: Kidney Education Lifestyle Application
NKF KDOQI: National Kidney Foundation Kidney Disease Outcomes Quality Initiative
RCT: randomized controlled trial
