# Supplementary figures and images for: Adequate Management of Phosphorus in Patients Undergoing Hemodialysis Using a Dietary Smartphone App: Prospective Pilot Study
Source: JMIR Form Res. 2021 Jun 1;5(6):e17858. doi: 10.2196/17858 (PMC8207257; doi:10.2196/17858)

The dialysis diet and fluid non-adherence questionnaire (DDFQ)


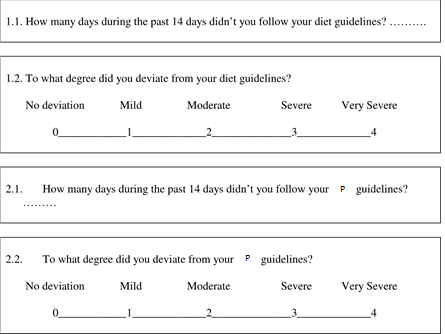

Supplement: Multimedia Appendix 1 [file formative_v5i6e17858_app1.docx]
