# Supplementary material for: Adequate Management of Phosphorus in Patients Undergoing Hemodialysis Using a Dietary Smartphone App: Prospective Pilot Study
Source: JMIR Form Res. 2021 Jun 1;5(6):e17858. doi: 10.2196/17858 (PMC8207257; doi:10.2196/17858)
Supplement: Multimedia Appendix 2 [file formative_v5i6e17858_app2.pdf]

Questionnaire to evaluate Kidney Dialysis Patients' knowledge on Kidney Diseases, Diet and Phosphate Binders

Place a circle around the correct answers for each question (There can be more than one answer)

**1.** Which of the following problems may result from high phosphorus in blood?	• Liver disease<br>• Cardiac disease<br>• Bone and joint disease<br>• High blood pressure
**2.** What happens when phosphorus levels are high in blood?	• Calcium will be pulled out of bones<br>• Osteodystrophy<br>• Calcium levels in blood will drop<br>• All of the above
**3.** What would high blood phosphorus level lead to?	• Heart arrhythmia<br>• Muscle cramp<br>• Dizziness<br>• Itchy skin
**4.** Why do you need to avoid all food items that are rich in phosphorus?	• There is no reason<br>• Because the liver is incapable of removing the excess phosphorus from the blood<br>• Because the kidney is incapable of removing the excess phosphorus from the blood<br>• Food items rich in phosphorus should consumed abundantly.
**5.** Which of the following foods contain high amounts of phosphorus?	• Sesame seeds, sesame paste, halawa<br>• Nuts & seeds<br>• Organ meats, Liver, sausage<br>• Lentils, chick peas, white kidney beans<br>• All of the above
**6.** What are phosphate binders?	• Medicine that decreases the absorption of potassium from food (in stomach) to the blood<br>• Medicine that decreases the absorption of calcium from food (in stomach) to the blood<br>• Medicine that decreases the absorption of phosphorus from food (in stomach) to the blood<br>• Medicine that decreases the absorption of magnesium from food (in stomach) to the blood
**7.** When is the right time to take phosphate binders?	• Between meals<br>• 8:00 – 13:00 – 18:00<br>• With every time I eat a meal<br>• Before every meal
**8.** Controlling an adequate blood phosphorus depends on: :	• The dialysis process<br>• A low phosphorus diet<br>• Phosphate binders<br>• The doctor<br>• All of the above
**9.** Which of the following food contain low amounts of phosphorus?	• Bread sticks without sesame seeds<br>• Sardine<br>• Honey, Apple jam, quince jam<br>• Coffee mate<br>• Brown bread

**10.** Which of the following drinks contain low amounts of phosphorus?	• Pepsi/Cola<br>• Lemonade<br>• Seven Up/Sprite<br>• Crush / Miranda<br>• Red Bull – Mountain Dew<br>• Coffee – Nescafe<br>• Tea (not dark color), Mint tea, Anise tea
**11.** Which of the following sweets is poor in phosphorus?	• Rice milk, pudding (made from milk) Custard,<br>• Jell-O<br>• Sorbet<br>• "Kunafa" with cheese (Arabic sweet)<br>• Chocolate – Chocolate cake<br>• Biscuits<br>• Milk based ice-cream
**12.** Which of the following food items are rich in phosphorus?	• String beans or green beans<br>• Milk and yogurt<br>• White bread and rice<br>• Tomatoes
**13.** Which are optimal ways to control an adequate blood phosphorus level?	• Take phosphate binders regularly<br>• Commitment to a low phosphorus diet<br>• Starving oneself
**14.** Which of the following food items are rich in phosphorus?	• Full fat milk and yogurt<br>• Low fat milk and yogurt<br>• Fat free milk and yogurt<br>• All have the same quantity
**15.** What is the optimal level for blood phosphorus?	• Higher than 8 mg/dl<br>• Less than 6 mg/dl<br>• Higher than 11 mg/dl<br>• Less than 1 mg/dl
**16.** Which food groups from the following are rich in phosphorus?	• Fruits<br>• Legumes: Lentils, chick peas, white kidney beans<br>• Nuts & seeds<br>• Vegetables<br>• Carbohydrates: bread, rice, pasta<br>• Dairy products: yogurt, milk, cheese
**17.** Which of the following pills are phosphate binders?	• Caltrate<br>• One Alpa<br>• Renagel<br>• Aspirin
**18.** Who is responsible in controlling your phosphate blood levels?	• The doctor<br>• The nurse<br>• You<br>• The dietitian

Thank you
